# Ambient biomass smoke and cardio-respiratory hospital admissions in Darwin, Australia

**DOI:** 10.1186/1471-2458-7-240

**Published:** 2007-09-13

**Authors:** Fay H Johnston, Ross S Bailie, Louis S Pilotto, Ivan C Hanigan

**Affiliations:** 1 Menzies School of Health Research, Charles Darwin University, Darwin, Australia; 2 School for Environmental Research, Charles Darwin University, Darwin, Australia; 3 Faculty of Medicine, University of New South Wales, Sydney, Australia

## Abstract

**Background:**

Increasing severe vegetation fires worldwide has been attributed to both global environmental change and land management practices. However there is little evidence concerning the population health effects of outdoor air pollution derived from biomass fires. Frequent seasonal bushfires near Darwin, Australia provide an opportunity to examine this issue. We examined the relationship between atmospheric particle loadings <10 microns in diameter (PM_10_), and emergency hospital admissions for cardio-respiratory conditions over the three fire seasons of 2000, 2004 and 2005. In addition we examined the differential impacts on Indigenous Australians, a high risk population subgroup.

**Methods:**

We conducted a case-crossover analysis of emergency hospital admissions with principal ICD10 diagnosis codes J00–J99 and I00–I99. Conditional logistic regression models were used to calculate odds ratios for admission with 10 μg/m^3 ^rises in PM_10_. These were adjusted for weekly influenza rates, same day mean temperature and humidity, the mean temperature and humidity of the previous three days, days with rainfall > 5 mm, public holidays and holiday periods.

**Results:**

PM_10 _ranged from 6.4 – 70.0 μg/m^3 ^(mean 19.1). 2466 admissions were examined of which 23% were for Indigenous people. There was a positive relationship between PM_10 _and admissions for all respiratory conditions (OR 1.08 95%CI 0.98–1.18) with a larger magnitude in the Indigenous subpopulation (OR1.17 95% CI 0.98–1.40). While there was no relationship between PM_10 _and cardiovascular admissions overall, there was a positive association with ischaemic heart disease in Indigenous people, greatest at a lag of 3 days (OR 1.71 95%CI 1.14–2.55).

**Conclusion:**

PM10 derived from vegetation fires was predominantly associated with respiratory rather than cardiovascular admissions. This outcome is consistent with the few available studies of ambient biomass smoke pollution. Indigenous people appear to be at higher risk of cardio-respiratory hospital admissions associated with exposure to PM10.

## Background

Short term associations between cardiovascular and respiratory hospital admissions and particulate air pollution have been demonstrated in many settings around the world, including multi-city studies in Europe, North America and Australia [1-5]. In most of these settings the main source of particles is fossil fuel combustion by industrial plants and transport although dust and biomass combustion can also make important contributions. An important gap in the currently available evidence concerns the roles of different sources of particles in contributing to ill health [6]. Although indoor biomass smoke is well recognised as a major cause of death and illness in developing countries [7] there is little evidence about the relative population health impacts of biomass combustion compared with other sources of airborne particulates [8]. However biomass combustion is becoming increasingly important as a source of ambient air pollution. The use of wood and other biomass fuels increased faster than overall energy demand in North America during the 1990s [9]. This has been attributed to its lower cost, renewable nature and a perception that wood smoke may be less harmful that exhaust from fossil fuel combustion [8]. Additionally there is a world wide increase in severe vegetation fire events associated with climate change and shifts in population settlement patterns [10,11]. The smoke from such fires has the potential to travel vast distances and affect major population centres far from the fires [12]. The increase in wild fires has prompted an increase in deliberate landscape burning to reduce fuel loads and avert major disasters but this practice has become increasingly controversial as the adverse health effects of particulate air pollution become more widely known [13]. While it has been argued that deliberate population exposure to smoke from management fires is justifiable to prevent large wild fires, there is an immediate need for evidence concerning the public health risks or potential benefits of various burning regimes [14].

The city of Darwin, northern Australia, provides a useful setting to examine the population health impacts of outdoor air pollution from biomass combustion. Here, approximately 95% of particulate pollution is derived from fires in the surrounding savanna, which cause a smoke haze of variable severity over the city for up to eight months each year [15]. Of the 110,000 residents of the city 11,500 (approximately 11%) are Aboriginal and 25,000 less than 15 years of age [16]. The relatively high proportion of indigenous Australians provides an opportunity to examine the differential impact of ambient air pollution in this population subgroup. Aboriginal Australians have a disproportionate burden of social disadvantage, diabetes, and chronic heart and lung conditions, all of which modify the effect of air pollution on health [17-23]. Examination of the magnitude of the likely disproportionate impact of air pollution in this group has been identified as an area of research priority by Australia's Environment Protection and Heritage Council [24].

We examined the relationship between atmospheric particle loadings 10 microns or less in diameter (PM_10_) and hospital admissions for respiratory and cardiovascular conditions for the three fire seasons (April to November) of 2000, 2004 and 2005. Air quality was not measured during 2001–2003.

## Methods

### Study design

We used a case-crossover design, in which each case is their own control [25]. Comparison of environmental data is made between the day each case was admitted to hospital, and several referent days on which they were not admitted. Measured and unmeasured individual variables such as age and smoking status are controlled by this design. The referent days were selected from the same month and year and matched by day of week of the admission. This time-stratified method of selecting comparison days has been recommended as it ensures unbiased conditional logistic regression estimates and avoids bias resulting from time trends in the environmental exposures being examined [26].

### Exposure measures

During 2000 PM_10 _was measured using a Rupprecht and Patashnick Tapered Element Oscillating Microbalance (TEOM) series 1400a. These data were validated by comparison with filter collections using an Ecotech MicroVol aerosol sampler with a 10 micron size selective inlet [15]. During 2004 and 2005 we used a Rupprecht and Patashnick Partisol plus model 2025 air sampler which provided 24 hour filter collections of PM_10 _(μg/m^3^) that were subsequently weighed on a mass balance. These data were validated by inter-laboratory comparison of gravimetric analyses conducted with the Marine and Atmospheric Research division of Australia's Commonwealth Scientific and Industrial Research Organisation (CSIRO). Our monitoring site was located close to the main residential areas. Particulate air pollution in Darwin has been demonstrated to be regional with high correlations between monitors located up to 25 km apart [27].

Daily meteorological data were provided by the Bureau of Meteorology and weekly consultation rates for influenza-like illness were provided by the Northern Territory (NT) Department of Health and Community Services from data routinely gathered from sentinel general practitioners.

### Outcome measures

Hospital admission data were collected by the Royal Darwin Hospital (RDH), the single major public hospital and referral centre for the northern half of the Northern Territory. At discharge from hospital, separation diagnoses are assigned according to the International Classification of Diseases version 10 (ICD10) [28]. De-identified emergency admissions data for 2000, 2004 and 2005 with a principal ICD10 diagnosis code for respiratory and circulatory conditions were extracted from the Northern Territory Government database (Table 1). These included patients admitted through the hospital's emergency department or by direct arrangement with private doctors. Each admission included details of date of birth, gender, ethnicity, ICD principal diagnosis code, occupation, place of residence, dates of attendance, admission and discharge and a unique identifier. These data were cleaned by identifying gaps and errors in the data extraction process and by finding and eliminating duplicate records. Those whose primary residential address was not in Darwin were excluded. The excluded group comprised residents of rural communities adjacent to Darwin, remote towns and Indigenous communities within the NT and interstate travellers. We included the first admission only for each episode of illness by excluding readmissions within 4 weeks of discharge.

**Table 1:** Clinical conditions and ICD codes examined for admissions to Royal Darwin Hospital, 1 April – 30 November in 2000, 2004 and 2005 (N = 724 days)

**Clinical group**	**ICD10 codes**	**Number of Admissions**	**Number Indigenous**	**Number <15 years old**
**Respiratory conditions – all**	**J00–J99**	**1474**	**384**	**548**
Asthma	J45–46	253	65	149
Chronic obstructive pulmonary disease	J40 – J44	305	76	0
Respiratory infections	J00–J22	778	218	378
**Cardiovascular conditions – all**	**I00 – I99**	**992**	**186**	**12**
Ischaemic heart disease	I20 – I25	422	81	0

### Data analysis

Respiratory and cardiovascular admissions were described according to Indigenous status and specific diagnostic groups for which associations with particulate air pollution have been previously documented. We used conditional logistic regression models to calculate odds ratios (OR) and 95% confidence intervals (CI) for hospital admission in relation to variation in PM_10 _adjusted for weekly influenza rate, days with rainfall > 5 mm, same day mean temperature and humidity, the mean temperature and humidity of the previous three days and public holidays. These potential confounders were chosen a priori and included in all models. School holidays were additionally included as a dummy variable for all respiratory conditions, asthma, and respiratory infections as these groups included a substantial proportion of children. (see Table 1.)

### Ethical approval

Ethical approval for this research was granted by the joint Human Research Ethics Committee of the Northern Territory Government and the Menzies School of Health Research (03/67), and the Human Research Ethics Committee of Charles Darwin University (H0369).

## Results

Environmental conditions and weekly influenza rates during the study period are summarised in Table 2. PM_10 _levels fluctuated throughout each dry season and often climbed or fell by 15–20 μg/m^3 ^over periods of just a few days. Australia's national air quality target for PM_10 _of 50 μg/m^3 ^was exceeded a total of seven times throughout the study period.

**Table 2:** Summary of meteorology data, PM_10 _and influenza consultation rates in Darwin, 1 April – 30 November in 2000, 2004 and 2005 (N = 724 days)

**Variable**	**Min**	**10^th ^percentile**	**25^th ^percentile**	**50^th ^percentile**	**75^th ^percentile**	**90^th ^percentile**	**Max**
Daily temperature °C	19	24.1	25.6	28.9	28.9	29.9	31.9
Daily relative humidity (%)	22	49.9	61.4	67.4	72.1	76.5	91.4
Daily precipitation (mm)	0	0	0	0	0	1.2	123.4
PM_10 _(μg/m^3^)	1.1	10.3	13.6	17.4	22.3	27.7	70.0
Influenza (weekly GP diagnoses per 1000 consultations)	0	4.3	6.9	10.9	18.2	25.7	61.9

**Figure 1:**
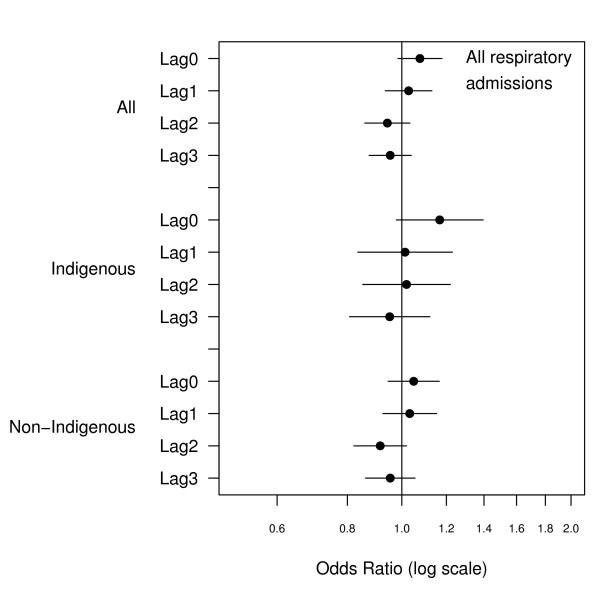
Adjusted odds ratios and 95% confidence intervals for hospital admissions for all respiratory conditions per 10 μg/m^3 ^rise in PM_10 _for the same day and lags up to 3 days.

**Figure 2:**
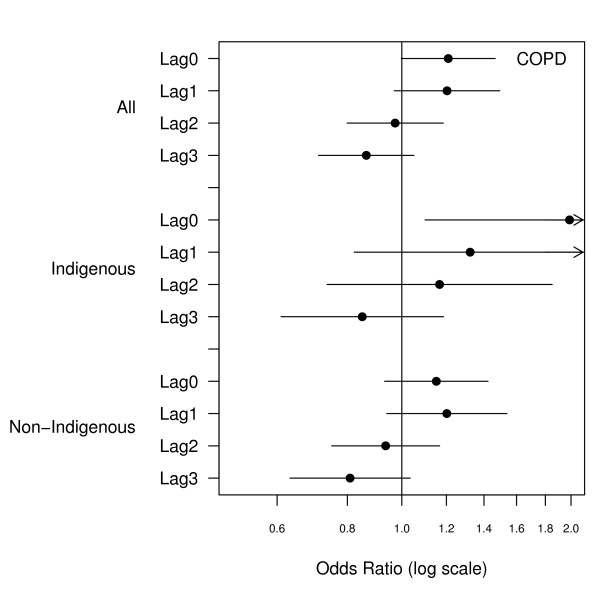
Adjusted odds ratios and 95% confidence intervals for hospital admissions for chronic obstructive pulmonary disease per 10 μg/m^3 ^rise in PM_10 _for the same day and lags up to 3 days.

**Figure 3:**
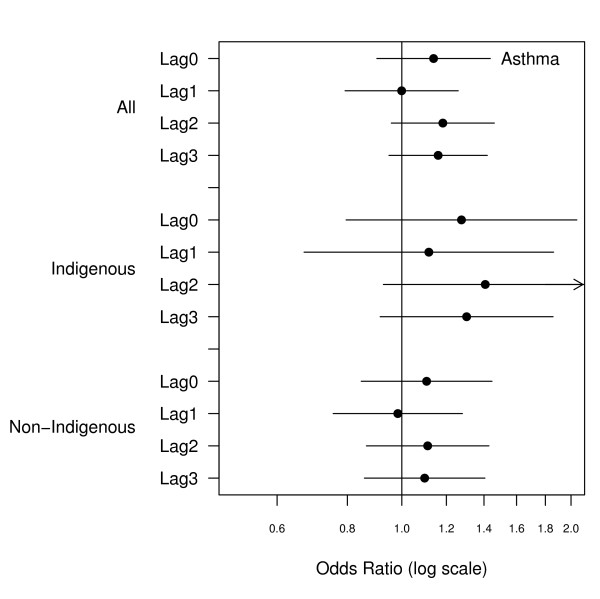
Adjusted odds ratios and 95% confidence intervals for hospital admissions for asthma per 10 μg/m^3 ^rise in PM_10 _for the same day and lags up to 3 days.

**Figure 4:**
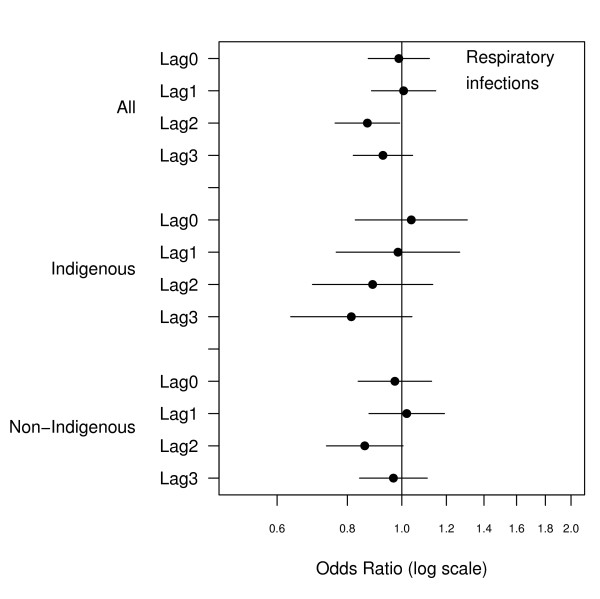
Adjusted odds ratios and 95% confidence intervals for hospital admissions for respiratory infections per 10 μg/m^3 ^rise in PM_10 _for the same day and lags up to 3 days.

**Figure 5:**
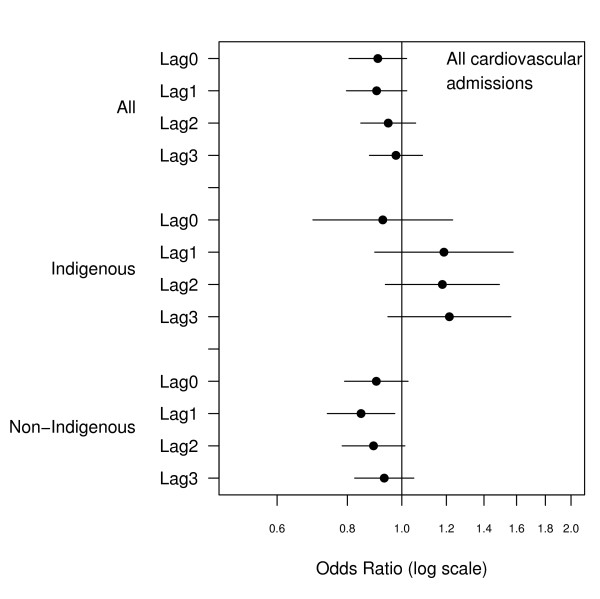
Adjusted odds ratios and 95% confidence intervals for hospital admissions for cardiovascular conditions per 10 μg/m^3 ^rise in PM_10 _for the same day and lags up to 3 days.

**Figure 6:**
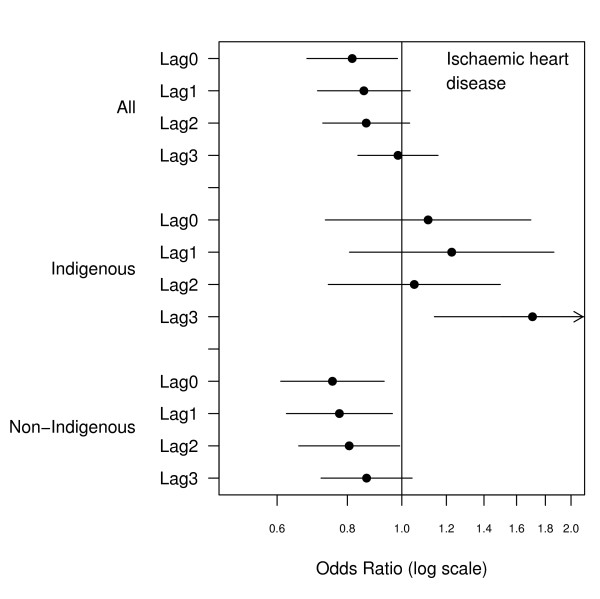
Adjusted odds ratios and 95% confidence intervals for hospital admissions for ischaemic heart disease per 10 μg/m^3 ^rise in PM_10 _for the same day and lags up to 3 days.

There were 2466 emergency admissions examined of which 23% were for Indigenous people. The number of admissions by ethnicity and clinical grouping are summarised in Table 1. The relationship between hospital admissions and PM_10 _for the same day and lags up to three days are presented in Figures 1, 2, 3, 4, 5, 6. Bordering on statistical significance, admissions for all respiratory conditions were positively associated with 10 μg/m^3 ^increases in PM_10 _from bushfires (OR 1.08 95%CI 0.98–1.18) with a larger magnitude in Indigenous people alone (OR1.17 95% CI 0.98–1.40). Conditions with the greatest positive associations were chronic obstructive pulmonary disease (COPD) (OR1.21 95%CI 1.0 – 1.47), asthma (OR1.14 95%CI 0.90 – 1.44) and asthma and COPD combined (OR 1.19 95%CI 1.03 – 1.38). The effect sizes were greater in Indigenous people, particularly those admitted for COPD whose odds of admission approximately doubled with each rise of 10 μg/m^3 ^in ambient PM_10_. (OR 1.98 95%CI 1.10–3.59). No association was observed between PM_10 _and respiratory infections.

There was no association between cardiovascular admissions in total and same day PM_10_, or lags of up to 3 days but among Indigenous people there was a positive non significant association at lags of 1 to 3 days. For ischaemic heart disease (IHD), there was a significant same day negative association (OR 0.82 95%CI 0.68–0.98) overall and in non-Indigenous people (OR 0.75 95%CI0.61–0.93). In contrast Indigenous people had a positive association that reached statistical significance at a lag of 3 days (OR 1.71 95%CI1.14–2.55).

## Discussion

We have described positive associations between particulate air pollution derived from vegetation fires and admissions to hospital for several respiratory conditions. Effect estimates were greatest for chronic lower respiratory conditions with Indigenous people being at highest risk. However, for cardiovascular conditions the associations were either negative or absent except in the Indigenous subpopulation, for which associations tended to be positive.

Our findings of predominantly respiratory impacts of air pollution from bushfires are consistent with the few available studies concerning attendances to health facilities associated with ambient biomass smoke. Several studies have documented rises of 30 –160% in attendances for respiratory conditions during episodes of poor air quality due to wildfires [29-32]. Additionally, two time-series studies have examined hospital admissions for both respiratory and cardiovascular outcomes in association with source-specific particulates generated by vegetations fires. Morgan et al compared the impact PM_10 _attributable to bushfires with PM_10 _attributable to all other sources in a hospital admissions series in Sydney from 1997 – 2001. While they found associations between cardio-respiratory admissions and PM_10 _from all sources, the principle associations observed in relation to bushfire generated PM_10 _was with respiratory rather than cardiovascular outcomes [33]. Mott et al examined cardio-respiratory admissions in Malaysia during severe forest fires in 1997 and also noted that admissions for respiratory, particularly asthma and COPD, rather than cardiovascular admissions were primarily affected by particulate levels [34]. This contrasts with many studies and meta-analyses of urban air pollution which have consistently demonstrated small positive associations with a range of both respiratory and cardiovascular outcomes including cerebrovascular diseases, IHD, heart failure and cardiovascular admissions overall [35,36]. Morgan et al postulated that association with cardiovascular morbidity from biomass smoke could be driven by PM_2.5 _a smaller size class fraction of particulates rather than PM_10 _the principal exposure measure for vegetation fire smoke reported in the above studies. This was based on their observation of an association between IHD admissions and bushfire derived particulates as measured by BSP, a measure of light scatter which is better correlated with PM_2.5 _than PM_10_. However this is a less likely explanation in our region where previous studies have demonstrated a very high correlation (r^2 ^= 0.81) between PM_10 _and PM_2.5 _[37].

Similarly, our findings of relatively large associations with respiratory outcomes are consistent with other studies of pollution from wildfires and biomass derived particulates. We observed increases of approximately 8% for all respiratory admissions, 20% for COPD and 13% for asthma admissions with incremental rises of 10 μg/m^3 ^of PM_10_. These were of similar magnitude to estimates reported by Mott et al for the South East Asian fires of 1997 and also to an earlier study of bushfire smoke and asthma attendances conducted in Darwin [32,34]. These findings are also supported by a recent study from Brisbane, Australia, which directly compared the association between bushfire and non-bushfire derived particulates on total respiratory hospital admissions excluding influenza [38]. That study analysed the PM_10 _distribution as a three-level factor with levels defined as low (<15 μg/m^3^), medium (15–20 μg/m^3^) and high (>20 μg/m^3^). They found that for an increase in same-day PM_10 _from low to high there was an increase in the relative risk for total respiratory hospital admissions of 19% (95%CI: 9%, 30%) whereas on non-bushfire days the associated increase was 13% (95%CI: 6%, 23%). Similarly Morgan et al reported 3.8–5% increases in association with bushfire derived particulate matter (PM) for COPD and asthma in Sydney, while not finding any association between these outcomes and PM from other sources [33]. All these estimated associations for biomass derived PM are well above those reported from meta-analyses of studies conducted in large cities of Europe and the USA that are of the order of 1–2% for all respiratory admissions and 1.5% for COPD and asthma in association with 10 μg/m^3 ^increases in PM_10 _[1,2,4]. They fit the pattern of a recent review of studies of PM_10 _and asthma that found greater relative risks documented in studies for which wood combustion was considered to be a major source of particulate matter [39].

While there appears to an emerging pattern of relatively greater respiratory and lesser cardiovascular adverse effects associated with particulates derived from vegetation fires compared with non-biomass sources, the available evidence is limited and further research is required to investigate this hypothesis. The relatively big effect sizes we observed could have other explanations. Darwin has relatively uniform population exposure from source-specific particulates [27], a single major hospital and excellent data collection systems in place, all of which reduce the risk of misclassification bias in the exposure and outcome measures compared with large cities with considerable regional variation in pollution levels and multiple health services. However, our relatively small population inevitably limits the precision of our point estimates as evidenced by wide confidence intervals.

The high proportion of Indigenous admissions is unlikely to have contributed to our higher effect estimates as the results for non-Indigenous admissions were very similar to the overall findings. However it is notable that the size of the associations between PM_10 _and admissions for all respiratory conditions, COPD and asthma were all more than double in Indigenous people. Additionally, and in contrast to non-Indigenous people, we observed a positive association with cardiovascular admissions in this group. While admission numbers were relatively small, and associations did not achieve statistical significance, it is clear from our findings that Indigenous people are at greater risk from ambient air pollution. This cannot be explained by differences in exposure, or individual factors such as smoking or socio-economic status as we controlled for these factors in the design of the study. It is more likely to reflect the greater burden of chronic cardio-respiratory diseases among Indigenous people, placing them at higher risk from environmental hazards such as air pollution [22,24]. Understanding the differential effects of air pollution in more vulnerable groups of people is important for determining public health policy such as the setting of air quality guidelines.

This study also highlights the public health implications of land management practices in countries with fire prone vegetation. Studies of severe air pollution generated by intense, uncontrolled fires in the USA and South East Asia have clearly demonstrated an association with serious outcomes such as hospital admissions and deaths [14]. Indeed, a recent economic analysis of a Canadian forest fire that burned for just 5 days, estimated that the health cost of the fire amounted to approximately $12 million, largely due to the premature mortality caused by air pollution. These costs were similar the estimated cost of timber losses, and greatly exceeded the costs of containing the fire [40]. There is no doubt that the prevention of such large fires is of high priority and the public health risks from smaller fuel reduction burns should be evaluated in this context. Our findings suggest that the health impacts of lower levels of pollution as observed during our study and frequently generated by deliberate burns also require serious consideration. For example, prescribed fires should be explicitly managed to minimize pollution over urban areas and be accompanied by public health advisories to reduce the impact on people at higher risk.

## Conclusion

PM_10 _was predominantly associated with respiratory rather than cardiovascular admissions in this setting where the vast majority of particulates are derived from vegetation fires. This pattern of results is in keeping with findings from the few other similar studies that have been reported. Adverse health associations were identified at relatively low levels of pollution, a result that has particular relevance for land and fire management practices world wide. Indigenous Australians are at greater risk of harm from particulate air pollution.

## Competing interests

The author(s) declare that they have no competing interests.

## Authors' contributions

FHJ conceived of the study, conducted the analysis and drafted the manuscript. RSB and LSP provided guidance on epidemiological methods and helped draft the manuscript. IH assisted with data analysis and helped draft the manuscript. All authors read and approved the final paper.

## Pre-publication history

The pre-publication history for this paper can be accessed here:


